# “I feel better as a parent” – summer programs help support children’s health behaviors and parent wellbeing

**DOI:** 10.21203/rs.3.rs-9125988/v1

**Published:** 2026-04-23

**Authors:** Emily K. Eglitis, Meghan Savidge, Elizabeth L. Adams, Michael W. Beets, Andrew T. Kaczynski, Brian Chen, Bridget Armstrong, Sarah Burkart, Keagan P. Kiely, James W. White, Olivia Finnegan, Hannah Parker, Griffin A. T. Randolf, Carol Maher, R. Glenn Weaver

**Affiliations:** Adelaide University; University of South Carolina; University of South Carolina; University of South Carolina; University of South Carolina; University of South Carolina; University of South Carolina; University of South Carolina; University of South Carolina; University of South Carolina; University of South Carolina; University of South Carolina; University of South Carolina; Adelaide University; University of South Carolina

**Keywords:** Summer holidays, summer programs, parents, families

## Abstract

**Background:**

Summer holiday programs offer a promising solution to prevent unhealthy changes in health behaviors often experienced by children during the extended break from school. However, not all families are able to access summer programs. This qualitative study explored parents’ experiences of the summer holiday period and their perceptions of receiving free summer programs to identify potential benefits, facilitators, and challenges to access.

**Methods:**

Parents (N=24: 100% female, 63% Black) of families randomized to receive free summer programming or experience “summer as usual” (control) were interviewed at the conclusion of summer 2024. Parents of “high-attenders” (N=8), “low-/non-attenders” (N=8) and controls (N=8) were recruited to participate in a semi-structured interview. Participants were asked about general summer experiences and benefits/challenges of the free summer program. Interviews were audio recorded, transcribed and coded. Thematic analysis was conducted with themes compared across attendance groups.

**Results:**

Parents described variability in their summer holiday experiences compared with the school year. Themes included family functioning, daily routines, and children’s health behaviors. Families with greater work flexibility and financial resources more often reported psychosocial benefits, including reduced stress and increased family time. Families with fewer financial or social support reported greater stress managing childcare demands and activity costs. Control-group parents particularly described stress juggling family roles while trying to provide healthy and enjoyable summer experiences. Summer programs were perceived as providing structured, active, and socially engaging environments that supported children’s physical, social, and emotional wellbeing while meeting parents’ childcare needs. Cost remained a significant barrier. Families’ values, needs, and practical constraints shaped engagement decisions. Free programs improved access and reduced financial distress. Facilitators to engagement included program design, content, and delivery features.

**Conclusion:**

Families with stronger financial and social support were better able to offset the increased demands of the summer holidays. Summer programs functioned as a social safety net and provided opportunities for physical activity, social connection, and cognitive engagement while supporting family wellbeing. Ensuring equitable access to summer programs represents a practical and scalable opportunity to support children and families. Future research should explore sustainable funding models and fee structures that promote participation while remaining equitable.

**Trial registration:**

NCT05880901

## Background

In addition to providing education for children, school plays an important role in supporting children’s health and wellbeing. The structure of the school day helps to regulate children’s health behaviors ^1^. Schools also provide childcare, meals, and opportunities for social connection. In this way, schools serve a broader supportive function for families. Summer is the longest consecutive period children spend away from these supports which present significant challenges for families ^2-4^. Away from school, children are often less active, spend more time sedentary and on screens, and display less healthy sleep and diet behaviors which can negatively impact academic outcomes and body weight ^5-11^. Families with fewer financial or social resources may struggle to compensate for the loss of school-based supports and are therefore more likely to experience these adverse effects ^7,12^. Therefore, interventions that provide structure and replicate similar supports of the school environment, such as structured summer programs, represent a promising strategy to mitigate declines in children’s health and wellbeing over summer while also supporting families more broadly.

Summer programs, such as day-camps, provide fun, enriching activities delivered across a typical school day for several weeks during summer. Similar to school, these programs provide structure that can support healthy behaviors and limit excessive weight gain ^13,14^. Evidence indicates that structured summer programs can increase moderate-vigorous physical activity (MVPA) and reduce sedentary behavior and screen time ^13,15^. There is also emerging evidence that participation may support children’s mental health ^16^. Children experiencing socioeconomic disadvantage appear to be at greater risk of health declines over summer^7^ and may therefore have the most to gain from participation in structured programs. However, families living on low incomes often face substantial barriers to access, particularly due to the high financial cost of attendance^17^. As a result, unequal access to summer programming may inadvertently widen disparities in key health outcomes, including overweight and obesity prevalence.

Although structured summer programs demonstrate benefits for child health outcomes, the literature has primarily focused on quantitative changes in physical activity and adiposity. Less attention has been paid to families’ lived experiences of summer, the contextual challenges they face, and the factors that influence participation in available programs. In particular, limited research has examined why some families attend consistently, others disengage, and some do not participate at all, even when programs are offered at no cost. Without this understanding, well-intentioned interventions may fail to reach those most at risk or may inadvertently widen health disparities. Evaluating interventions designed to support children experiencing disadvantage, such as free summer programs, requires consideration not only of effectiveness but also of equitable participation. Within the RE-AIM framework, population impact depends in part on Reach – who participates, who does not, and whether participants are representative of those most at risk^18,19^. Without this understanding, even well-designed interventions may fail to reach children who stand to benefit most or may inadvertently widen health disparities if barriers to engagement remain unaddressed.

Accordingly, this qualitative study sought to explore parents’ experiences of summer holidays and their perceptions of summer programs, with particular attention to factors influencing participation. By comparing parents of high attenders, low/non-attenders, and families experiencing summer as usual, the study aimed to identify the challenges families face during summer, the perceived benefits and burdens of program participation, and the barriers that may limit equitable access. The research questions were:

1. What changes do families experience between the school year and summer holidays, and how do they perceive those changes?
2. What are the perceived benefits and challenges of attending summer programs?

## Methods

### Ethics

Ethical approval for this study was obtained from the University of South Carolina Institutional Review Board Pro00125471. All participants provided informed consent prior to participation. Participants were informed that their participation was voluntary, their responses would be de-identified, there were no “right” or “wrong” answers and that they could withdraw at any time without consequence. At the conclusion of the interview, participants were provided a $30 gift card.

### Study design and participants

This qualitative study was embedded within “Beyond the Bell”, a larger, three-year randomized controlled trial evaluating the effects of free programming on children’s health and wellbeing, described elsewhere (registered May 2023 with ClinicalTrials.gov, registration number NCT05880901 ^20^). Briefly, children (5-12 years) from one school district in the southeastern United States which predominately served families with low incomes, were recruited. Families were randomized to receive free access to an existing summer day camp (intervention) which ran five days per week throughout summer and included structured activities including physical activity, enrichment, and meals or continued with “summer as usual” (control). Participants of this qualitative study were a sub-sample of parents (N=24: 100% female, 63% Black) recruited from the first year of the Beyond the Bell study. Recruitment occurred via a text message inviting them to provide their perspectives on their summer experiences with and without free summer programming. In total, n=27 of participants indicated their interest in the study but n=3 were unable to be contacted for the interview. This study is reported in accordance with the COREQ guidelines (Additional file 1) for qualitative studies, which are appropriate for use with interview data ^21^.

### Data collection

At the conclusion of summer 2024, parents were purposefully recruited based on their child’s summer program attendance. For intervention families, summer programs were provided at no cost. Groups consisted of “high-attenders” (N=8, ≥50% of offered program days were attended), “low-attenders” (N=4, <50% of offered program days were attended), “non-attenders” who were randomized to receive free access but did not attend (n=4) and control (N=8, “summer as usual”). Some control families chose to enroll their child in a summer program (attendance level not recorded) and pay the attendance fee.

Therefore, control-group children enrolled in a summer program may have had similar experiences to the intervention group, but only the control families experienced the financial burden of attending.

Parents participated in a semi-structured phone or in-person interview conducted by two members of the research team, both female public health doctoral students with didactic training, and/or direct prior experience in qualitative methodology (MS or EE) (August to October 2024). Both interviewers had an academic interest in children’s health and wellbeing and the potential role of structured summer programs. Reflexive discussions were undertaken during data collection and analysis to consider how the researchers’ perspectives and assumptions might influence interpretation. An interview guide (Additional file 2) was developed and pilot tested by the research team prior to data collection. Participants and interviewers were unknown to each other at the commencement of the study. Participants were informed that the purpose of the interview was to learn more about their summer and their experience with the study in order to understand how attending a free summer day camp impacted their child, themself and their family. Questions related to experiences of summer, the shift from the school term to summer and of summer programming (in general and related to free summer programming).

Interviews were conducted in two parts: interviews of high-attenders and control participants were conducted by MS (a US researcher with a nutritional background) face to face at the university research center (with an extra research assistant present) or individually via phone. Interviews of low- and non-attenders were conducted by EE (an Australian researcher with a physical activity background) via phone. Interviews lasted 30 to 45 minutes. All interviews were audio-recorded and transcribed verbatim using Otter.ai (Otter.ai, Mountain View, CA, USA), an automated speech-to-text transcription software. No repeat interviews were conducted, and no field notes were made. Transcripts were checked for accuracy by interviewers and research assistants. Transcripts were not reviewed by participants, and they did not provide feedback on the findings.

Sufficiency of information power was used to determine the conclusion of data collection ^22^. Toward the conclusion of analysis, when no new codes or themes emerged and themes were well developed, the research team agreed that the dataset was sufficient to answer the research questions and data collection was ceased.

### Data analysis

Guided by a phenomenological approach, the analysis focused on how participants perceived and made sense of their experiences. Inductive thematic analysis was used to systematically code the data and develop themes that reflected shared aspects of these lived experiences. Inductive thematic analysis is a flexible yet systematic approach not tied any specific theoretical framework ^23,24^. It involves familiarization with the data, generating initial codes, collating codes into themes, reviewing and naming themes and then producing a summary report ^23^. All interviews were analyzed and coded independently in duplicate (MS and EE) using Nvivo software (version 14; Lumivero, Denver, CO, USA). A constant comparison approach^25^ was used where theme generation was compared, consolidated and discrepancies agreed upon. Uncertainties were referred to the senior authors of the research team (GW and EA). A comparative analysis of themes was conducted (by EE) comparing general summer and summer program experiences across attendance groups (high-attenders, low-attenders, non-attenders, control).

## Results

### Sociodemographic data

Sociodemographic characteristics of the study participants is presented in Table 1. A coding tree was generated to depict the final hierarchical structure of the analysis, showing how inductively derived codes clustered into subthemes and themes (Additional file 3).

### Research Question 1: What changes do families experience between the school year and summer holidays and how do they perceive those changes?

Families had to plan for how children would spend their time at home over the summer. Children either spent their time either at home with their parents, with extended family or at a structured summer day camp. Families’ experiences of summer varied markedly by attendance group. Control families described the most disrupted summers. Their days were least structured with children spending long periods at home, often on screens, and experiencing considerable relaxation in sleep routines. Parents frequently adjusted work hours or took leave to supervise children, and boredom and financial strain were dominant challenges. Non-attenders had similar experiences,while low-attenders had a more mixed summer experience with partial program participation offering some structure. High-attenders, in contrast, had the most structured and manageable summers. Consistent program attendance helped maintain school-like routines, supported regular sleep patterns, reduced boredom and screen time, and enabled parents to sustain employment with minimal disruption. Their challenges centered on coordinating logistics, rather than a lack of care. Five main themes were identified related to families’ experiences and strategies for managing the shift from school to summer with considerable variation across families. The key themes were (1) Family functioning and roles, (2) Time-use and daily routines, (3) Health behavior shifts, (4) Financial challenges and (5) Psychosocial impacts. Themes are discussed here and summarized in Additional file 4.

#### Family functioning and roles

Family function was used to describe how the family operates to meet the emotional, social, physical, and developmental needs of its members. This theme includes the roles, communication patterns, problem-solving abilities, emotional support, and adaptability within the family. Parents described their family roles, such as primary caregiving, meal preparation, and managing household routines, as largely consistent across the school year and summer. However, the time demands associated with these roles often shifted. When formal childcare or camp arrangements were unavailable, such as for the control group or previous summers without free programs, parents spent considerably more time providing direct care and supervision. Many described heightened pressures during the summer months, as they sought to balance multiple, and often competing, demands (e.g., maintaining paid work, managing household responsibilities, and ensuring their children were engaged and cared for), which was particularly challenging for single parents or those without extended family support. High-attender families often relied upon summer programs for childcare support. Free summer programs provided welcome support for these families.

“It’s usually a big shift… I have to work, and so it’s kind of a really, really big shift… it’s hard because they don’t let you off just because your kids are out.”

(T19, high-attender)

For other parents with fewer competing demands the removal of school-related pressures, such as morning routines and homework, created opportunities to slow down and spend more quality time together. Families who could afford to have a primary carer at home over the summer holidays frequently described the summer as a welcome reprieve which enabled quality time, shared activities and family holidays:

“I love being with her… when she was younger, it would have been a relief just to get her out of the house… but I don’t feel that way now. Now, I love being with her.”

(T15, non-attender)

“(In summer) I don't have to, like, worry about picking her up from school. I don't have to worry about, hey, we got homework due. Let's cram it. And it's like, I can't really take the time that she needs, because (during the school year), I'm like, “Come on. Like, let's go. Let's go!”

(T17, control)

High-attenders noted strong parent benefits to summer programs, including reliable childcare, work productivity, reduced stress, and substantial financial relief. Among control families with primary carers at home, those able to afford fun, enriching, and engaging activities more consistently reported enjoying summer, whereas families with fewer resources described monotony and boredom arising from the lack of routines.

#### Time-use and daily routines

Time-use and daily routines refer to how families structured and organize children’s days, including the allocation of time to activities such as meals, sleep, hygiene, play, screen use, and organized programs. A continuum existed in the level of structure displayed, from highly maintained routines that prioritize predictability and continuity, to flexible or loosely structured days characterized by greater child autonomy and parental discretion.

Families commonly described summer as a period with fewer external structures, offering children more freedom while placing greater responsibility on parents to organize daily activities. Experiences varied considerably across households. High attendance necessitated consistent routines for meals, bedtimes, and hygiene, creating continuity and predictability:

“Our family routine is pretty much the same… we eat dinner together at the same time every day, bedtime at the same time, bath and showers at the same time.”

(T06, high-attender)

Low-attender families overall adopted a more relaxed approach. Some parents allowed children to take days off from the program to enjoy a break from routines and spend time at home, creating greater day-to-day flexibility in schedules. Others took weeks off at a time, usually for family holidays. While enjoying greater freedom from routines, parents often tightened routines in the lead up to school the school year:

“(Regarding routines,) I do give them a little more leniency for bedtime during the summer, although we do still try to keep the general routine there, because I don’t like having to re-train them back on the sleep routine for the school year.”

(T07, low-attender)

At the opposite end of the spectrum, some control-group parents described an almost complete loosening of structure, reflecting the unbounded nature of summer:

“I really don’t care what time she wakes up… or what time she goes to bed… she really doesn’t have a routine during the summer like she does during school.”

(T17, control)

Children’s preferences also influenced how time was spent. Some were content with unstructured relaxation (e.g., playing video games), while others sought stimulation and social activities.

“When they come home from (school) they, well, every child is different. So, it was different from (child’s name), what (sibling name). (Child’s name), he just, “yes, I'm home, I get to relax, I can play my video games.” And (sibling’s name) just like, “Yay, well, what can we do? What are we going to get into? Are we going to the beach”, things like that.

(T08, control)

Routines appeared strongest when families had structured activities and commitments outside of the home (e.g., parent work, child program attendance). Early in the summer holidays, short breaks from routines were welcomed as opportunities for rest and recovery from these external pressures. However, prolonged disruptions to routines often led to monotony, boredom and more sedentary behaviors.

#### Health behavior shifts

Health behavior shift refers to changes in children’s sleep, screen use, diet, and physical activity patterns that occur during summer in the absence of school-based structure. Parents described taking on increased responsibility for regulating children’s health behaviors during summer. Without the external structure of the school day, maintaining healthy habits required additional parental effort and intentionality. Many found this challenging, especially when affordable and accessible options for structured activity were limited. As one parent described her experience in previous summers:

“(Being home) triples the screentime. Even though my husband may be at home, he’s working from home… she is probably either watching TV, some YouTube or a tablet or something.”

(T12, high-attender)

Parents also expressed concern about the lack of opportunities for physical activity and social engagement when children spent long hours indoors or in sedentary care arrangements. One parent who usually relies on the children’s grandparents for childcare support over the summer holidays noted:

“We’ve noticed that there’s not a whole lot of activity, and it gets very boring for them … not being out with friends and doing activities and that sort of thing.”

(T07, low-attender)

Environmental factors such as heat and cost further limited families’ options for outdoor play:

“She (my daughter) wanted to do something this summer, but I really couldn’t find much… It’s super-hot – walking into an oven… we went to the park for a little bit yesterday. He (my son) was miserable.”

(T08, control)

Families consistently expressed a desire to keep their children physically active, socially engaged, and cognitively stimulated during summer. However, access to the time, financial resources, and support needed to facilitate these experiences varied. Across attendance groups, summer programs were widely recognized as fulfilling these roles. High-attenders in particular reported consistently positive experiences, emphasizing the benefits of high-quality activities, child enjoyment, social connection, and physical activity.

#### Financial challenges

Financial challenges refer to increased economic pressure on families, characterized by higher costs and time demands, sometimes alongside reduced capacity for income earning. Financial challenges necessitated decisions around family budgets, time trade-offs (e.g., increased work to pay for fun summer experiences) and financial sacrifices.

Many families reported heightened financial strain during the summer months compared to the school year, particularly related to the cost of food, childcare, and recreational or enrichment activities. With children spending more time at home, expenses often increased while access to affordable options for structured care remained limited. Control families identified cost as the main barrier preventing enrolment in structured programs, despite recognizing their value. Parents described their past experiences budgeting carefully and seeking out free or low-cost activities to keep their children occupied and engaged:

“And then, it makes it a little frustrating that, you know, you do want to spend more time with your kids in the summer, but you know, you gotta work and make money, you know… It is a challenge, to keep them occupied, they want to go places, things like that. I have to look for free things. Or like, I have to budget it in.”

(T16, high-attender)

Rising costs of everyday outings, such as visiting the zoo or going to the movies, were commonly cited as barriers to participation in family activities:

“They wanted to go to the zoo… it’s going to be $95 for us to get in the zoo? Ninety-five dollars! I said, oh no, we might have to wait. We can do a movie night or something, like here.”

(T08, control)

There was an identifiable pattern in responses related to financial challenges: families who had an adequate combination of extended-family support, financial means (e.g., a second income) and work flexibility (e.g., some teachers) were better equipped to cover childcare needs and provide enrichment activities for their children with at least manageable levels of financial pressure. For many families living on low incomes, financial pressures were a key feature of summer. For the majority of families in the intervention group, receiving the summer program at no cost was essential in enabling their participation; without it, most would have attended far less or not at all. These patterns highlight how financial pressures functioned drivers of psychosocial stress within families.

#### Psychosocial impacts

Psychosocial impacts refer to the emotional, mental, and social effects experienced by parents and families in response to situational demands and pressures. These impacts may include both negative and positive changes in wellbeing, reflecting how individuals and families perceive, cope with, and adapt to changing circumstances.

Parents described a wide spectrum of psychosocial experiences over the summer holidays, ranging from heightened stress to improved family wellbeing. For many, juggling work commitments with caregiving responsibilities created mental, social, and emotional strain. The lack of childcare options and increased time spent managing sibling dynamics or children’s behaviors contributed to feelings of anxiety and fatigue. One parent discussed her feelings prior to receiving free summer programming:

“My anxiety is a little bit more up than usual… you’ve got to figure out a way for them to be watched while you work.”

(T02, high-attender)

Another told us:

“It’s so hot, so we don’t get them outside to play as much… just inside time, maybe just a little bit more sibling fighting going on.”

(T09, high-attender)

One parent described feelings of guilt and concern that she has little choice other than for her child to spend long days alone as she is unable to provide many positive summer experiences:

“I try… I don’t want him to just be here all day by himself… I feel bad for him, that he has to spend the summer that way.”

(T23, control)

Conversely, families with greater flexibility in their time (e.g., stay-at-home parents, teachers) and the financial means to provide positive experiences valued the slower pace and opportunity for shared activities. They described the summer as a time of reduced pressure and increased enjoyment.:

“Summers are a little bit more relaxed for us… the schedule isn’t so tight… we’re able to just have, to me, more fun.”

(T13, control)

Free-programs enabled parents to continue to earn an income, while children enjoyed the physical and social benefits of program attendance. Time at home was therefore experiences as less stressful than the summer:

“I feel less stress during the summer… I don’t have to worry about schoolwork and grades and things like that.”

(T14, high-attender)

Overall, families’ psychosocial experiences of summer varied considerably and appeared to reflect the balance between competing stressors and available supports. Families with an adequate combination of financial means, extended-family support, and work flexibility were better able to manage childcare demands and provide enriching experiences with manageable levels of strain. In contrast, for many families living on low incomes, summer was characterized by persistent financial pressure, which shaped daily routines, stress, and overall family wellbeing and free programs provided significant relief. However, patterns were not clear according to attendance group because once the cost barrier was removed, other factors influenced program attendance.

### Research Question 2: What are the perceived benefits and challenges of summer programs

Benefits and challenges of summer programs were discussed broadly. Parents described a wide range of prior experiences with summer program models that differed in structure, duration, cost, and quality. While parents were asked specifically about free programming, child factors and parent benefits (described below) were consistently reported across both free and fee-based programs. Participation in any summer program requires effort from families, with program design and delivery features (e.g., affordability, accessibility, flexibility, content, cost) also acting as either facilitators or barriers to engagement. Cost was an important barrier which was addressed by free programming, but it was not the only challenge. Non-attenders were constrained by program fit, including lack of transport and child dislike of specific program features (large setting, bullying, preference for another program). Low-attenders described similar issues but also cited planned rest days, child allergies, or family travel that contributed to lower or irregular participation. Ultimately, families’ experiences of summer programs and their participation decisions were shaped by how well the perceived benefits of a program outweighed the overall burden of participation. Perceived benefits and challenges to summer programming are presented in Table 2 (detailed in Additional file 5).

#### Parent challenges

Parent challenges refer to the financial, logistical, and program-related difficulties parents experience when accessing or managing summer programs. These challenges are shaped by cost, availability, scheduling, and perceived program quality, and may differ depending on whether families have access to free or fee-based programs.

#### Financial challenges

While many parents valued summer programs for easing family and work pressures, they described significant financial and logistical challenges that influenced their ability to participate. The cost of summer programs emerged as the most pervasive barrier, shaping whether families could enroll their children, how often they attended, and which programs they chose. Parents, especially those with multiple children, described struggling to find affordable options:

“One of the biggest challenges this year was just finding affordable camps for the kids… even if they’re charging $100 per child a week, that’s $300 a week (for my children) and $1,200 a month.”

(T13, control – attended other program)

“It’s $75 a week… and some people might not think that’s a lot, but when you have other things, on top of stuff, I can’t do it.”

(T17, control)

Families with the fewest financial and social supports expressed the greatest need for summer programs, viewing them as essential for both childcare and children’s wellbeing. Free programs were described as transformative, reducing financial strain and allowing parents to direct income toward food, housing, or family activities:

“All we would have been doing was working for her to attend camp… it would have made it a lot more stressful with that aspect.”

(T12, high-attender)

“(Providing food is beneficial because) sometimes that’s the only way that certain kids are able to eat.”

(T19, high-attender)

#### Logistical challenges

Logistical barriers could also limited participation, even for free programs. Parents described the difficulty of coordinating transport, adjusting work hours, or managing options across the summer:

“(In previous summers) I had to find a cheer camp here for one week, a dance camp there for one week… they’re only 9 to 12, so now I’m running across town, drop you off and run back to pick you up.”

(T12, high-attender)

#### Program quality

Parents also noted that the quality and flexibility of programs mattered. Some camps were perceived as overly academic or insufficiently responsive to children’s individual needs, limiting their appeal:

“It’s more like they just sit in the classroom and do homework… basically like going to summer school.”

(T05, control – attended other program)

“It could be 20 people (staff) in there, but if they (the staff) don’t all know that these kids have this or that, then it doesn’t matter… they don’t have the education to help the kids.”

(T01, control)

Among families receiving free programs, low attendance occasionally occurred. This was typically due to child choice, medical conditions, family holidays, or the desire for rest rather than dissatisfaction:

“There were a couple days she just simply was tired and asked, ‘Can I just go to Grandma’s today?’… but she made good friends and it was overall beneficial.”

(T07, low-attender)

Overall, the cost, flexibility, and design of summer programs operated together to determine whether families could participate and benefit. For many, free or subsidized programs functioned as a critical safety net supporting not only children’s development but also parental wellbeing and family stability during the summer months.

#### Parent benefits

Parent benefits refer to the ways summer programs support parents’ capacity to manage time, work, caregiving, and family responsibilities, while contributing to reduced stress and improved family functioning. These benefits include practical relief, emotional reassurance, and greater confidence that children’s needs are being met in a safe, supportive environment.

Parents emphasized the important role that summer programs in general have played in alleviating many of the practical challenges associated with the school holiday period, especially when they were free. Programs provided reliable childcare that enabled parents to continue working, manage household responsibilities, and maintain more stable family routines. Parents described feelings of relief and reassurance knowing their children were safe and engaged while they fulfilled other responsibilities:

“Me having an opportunity to go to work without leaving them here by themselves all day. Me having peace of mind knowing that, where they’re at, they are safe. So, I think that’s the biggest thing.”

(T13, control – attended other program)

Beyond these logistical benefits, parents described positive flow-on effects to overall family functioning. Having children attend programs helped maintain structured routines and reduced sibling conflict by providing outlets for children’s energy:

“It benefits me in her general attitude… when the kids are more active, having a way to let out their energy… that energy, if it’s not let out in a productive way, they let out in destructive ways.”

(T07, low-attender)

Several parents expressed that programs eased fatigue and reduced the emotional strain of being the primary caregiver, giving them a sense of balance and time for rest or self-care:

“A little bit of peace during the day, of course. Not that he’s a bad child, not at all, but it’s nice to have a break.”

(T03, low-attender)

In some cases, access to affordable or free programs also provided financial relief and reassurance that children were being cared for in a stimulating environment:

“I’m very thankful. I’m very grateful. Because it’s hard out here. Everything’s getting expensive… it just makes me happy that she gets the opportunity to go to camp this year.”

(T16, high-attender)

Overall, free summer programs supported parents not only by easing time and financial pressures but also by enhancing family wellbeing, creating space for parents to manage competing responsibilities while feeling confident their children were safe, active, and happy.

#### Child factors

Child factors refer to individual child characteristics that influence how summer programs are experienced and perceived, including differences in needs, preferences, abilities, health considerations, and comfort in social or structured environments. The perceived success and suitability of programs depend on how well they accommodate these individual differences and support children’s engagement, wellbeing, and participation.

Parents consistently described structured summer programs as providing meaningful opportunities for their children to be physically active, socially engaged, and cognitively stimulated. Attending programs helped children spend less time on screen-based devices and more time participating in enjoyable, purposeful activities. Parents viewed these experiences as valuable for promoting both health and psychosocial wellbeing, noting that children who attended programs appeared happier, less bored, and more settled at home:

“The benefits for him are he’s staying active, he’s socializing… it tires him out, but it’s still good for him than being at somebody’s house just playing video games all day.”

(T14, high-attender)

Parents highlighted the diversity of activities, ranging from outdoor play to community excursions, that kept children engaged and exposed them to new experiences:

“He gets to play, go outside, interact with other kids… they go to the library, the movies, the splash pad… while if he’s just home, it’s just playing on a phone or tablet all summer.”

(T05, control – attended other program)

Social learning and interpersonal development were also perceived as major benefits. Programs gave children opportunities to form friendships, practice social skills, and gain confidence in navigating new environments:

“I just like the social part of it for him… learning how to be with people, learning how to deal with certain situations that he wouldn’t encounter at home.”

(T11, control – attended other program)

Despite these benefits, parents also described several child-related challenges that influenced attendance or enjoyment. Some children were resistant to attending, preferring the comfort of home or smaller, quieter environments. Others had medical conditions (such as severe allergies) or different social preferences that made participation more difficult. Managing multiple children with differing needs or interests sometimes added further complexity:

“(My daughter) loved it… (my son)’s my homebody. He did not want to do that.”

(T08, control)

“Sometimes he doesn’t want to go… he likes small settings… and my daughter’s opposite.”

(T04, non- attender)

Together, these accounts suggest that while structured programs offer substantial benefits for children’s health, learning, and wellbeing, their success depends on how well programs accommodate children’s individual differences, preferences, and comfort levels. The main features influencing engagement are summarized in Table 3. (The influence of family needs and program cost on summer program experiences is presented in Additional file 6).

## Discussion

This study examined parents’ experiences and perceptions of the summer holidays and explored how summer programs support the health and wellbeing of children and families. Key findings indicated families experienced the transition from school to summer in diverse ways. While family roles largely remained stable, the time required to fulfill household and caregiving responsibilities shifted in the absence of school-provided meals and childcare. Families with flexible employment, strong social networks, and adequate financial resources reported more positive experiences, characterized by reduced pressures and increased quality time. Conversely, families lacking these supports described summer as a period of heightened stress and frustration. Free summer programs mitigated these challenges, whereas high program costs either limited participation or imposed additional financial strain when caregivers were responsible for the program cost.

Parents perspectives in this study align with research that the school day provides beneficial structure that supports healthier behaviors in children ^12^, including additional functions such as meal provision and childcare. A key insight from this study was that the structure provided by school extended beyond school hours, increasing structure in the rest of the day too. For example, school days involved homework, extra-curricular activities, earlier wake-up times, and early morning or afternoon routines. Parents discussed managing school days with stricter bed-time routines and rules around discretionary time-use including screen time. While these rules and routines help families manage daily commitments, enforcing them can be burdensome and a source of stress, pressure, and fatigue for parents. Therefore, the start of the summer holidays represents a welcome reprieve from these pressures but over the course of summer, other pressures like meal provision, childcare, entertainment and opportunities for social connection accumulate.

Families’ experiences of summer differed greatly, as did levels of household rules and routines over the summer holidays. Household rules/routines reflected parents’ needs and work demands – parents who continued working over summer were bound to a more structured day than their children who experienced a drastic reduction in external structure. This mismatch was a frequent source of stress and tension, particularly for control group families. Participation in summer programs appeared to help restore alignment of family rules and routines, particularly sleep and wake times. When children were motivated to attend, they actively participated in family routines which was perceived to improve overall family functioning. These were important perceived benefit of summer programs. The greatest participation in free summer programming appeared to come from families where primary caregivers had work or study commitments. Families with less childcare need, for example where a caregiver was at home, participated less. Importantly, lower attendance was rarely attributed to dissatisfaction with the programs. Rather, it reflected family circumstances, competing opportunities, and children’s desire for unstructured time during summer.

Cost played an interesting role in participation. High cost was the most common factor reducing or preventing participation in summer programs. Making the program free enabled access for families on low incomes who otherwise would not have been able to afford it. However, making the program free did not necessarily promote high attendance levels. Without a financial investment, families did not “lose” anything by not attending. Some families reported that on days when children woke up and did not feel like attending, it was easier simply to stay at home. This is not ideal for program providers who need to organize staff and resources and inconsistent attendance is less likely to have the same benefits to children of consistent moderate or high attendance. It is also possible that the complete removal of cost reduced the overall perceived value of the program.

This study has several strengths. It is the first, to our knowledge, to explore family experiences engaging with summer programs, providing novel insights into how these programs support children’s and families’ wellbeing. The study employed a rigorous and systematic approach to analysis, including duplicate coding, collaborative theme development, and iterative review to enhance credibility and trustworthiness of findings. However, some limitations should be acknowledged. The study aimed to examine differences in experiences and perceptions by attendance level. Although we reached information saturation, we acknowledge that we were only able to recruit limited numbers of low- and non-attenders overall. They were likely the group who were the least engaged in the study and therefore may hold important perspectives, and a greater diversity in experiences that were not captured. It is also possible that the different interviewer who conducted the interviews for low- and non- attenders may have influenced the information volunteered by participants.

Future research should explore how different program funding and fee structures influence family engagement and child outcomes. While free programs reduce financial barriers, this study suggests that no-cost access alone does not guarantee consistent attendance or optimal engagement. Understanding the balance between affordability, perceived value, and commitment could help identify fee models that promote sustained participation while remaining equitable. Further investigation is also needed into sustainable funding mechanisms beyond research-supported programs. Studies examining how local, state, and federal governments can effectively invest in or subsidize summer programming would provide critical evidence to guide policy decisions and ensure that these programs remain accessible to families who benefit most from them.

## Conclusion

The experience of summer holidays differs widely between families. Those able to manage the absence of school-based supports often enjoy reduced stress, more quality family time, and opportunities for recreation and travel. Summer programs play an important role in supporting children’s healthy behaviors, social development, and emotional wellbeing during this period. While cost remains a major barrier to participation, it is not the only factor influencing engagement. Well-designed programs that are affordable, accessible, and offer safe, enriching, and flexible experiences can help families navigate the challenges of summer more positively. Future program models should consider both structural and contextual factors including affordability, flexibility, and meaningful content to ensure equitable access and sustained participation.

## Supplementary Material

This is a list of supplementary files associated with this preprint. Click to download.

- A5Benefitsandchallenges.docx
- A6Influenceofcost.docx
- A2Parentinterviewguide.docx
- A1COREQchecklist.docx
- A4Themesdetailed.docx
- A3Codingtree.docx

## Figures and Tables

**Table 1.** Socio-demographic characteristics of study participants (N= 24)

	Control (n=8)	SDC High-attender (n=8)	SDC Low/non-attender (n=8)
**Sex** (% female, n)	100% (8)	100% (8)	100% (8)
**Race** (n=)			
Black	5	5	3
Multiracial	1	0	3
White	2	3	2
**Ethnicity** (n=)			
Hispanic-Latino	2	0	0
**Income**			
Total Assistive Benefits* (n out of 4)			
0	1	2	2
1	3	1	0
2	2	5	3
3	0	0	3
4	4	0	0

Note:

* Total Assistive Benefits: the number of government assistance programs enrolled by the family (higher numbers indicate greater financial support as a proxy measure for lower income and include X, Y, Z).

**Table 2.** Summary of perceived benefits and challenges of structured summer programs

	Benefits	Challenges
**Parent factors**	Childcare: easier for parents to continue with paid employment and unpaid chores/tasks more easily, respite for primary caregivers Supported family functions (regulating daily routines, keeping kids more active) Reduced parent fatigue Financial relief (free programs)	Financial constraints (general programs) Logistical demands Perceived program quality and alignment with parent values
**Child factors**	Healthier behaviors (reduced screen time, increased physical activity) Learning: cognitively stimulating activities and environments Fun, enjoyment, improved mood and reduced boredom Social engagement	Program appealing/child resistance Medical conditions to manage (e.g., severe allergies) Managing children at different program locations

**Table 3:** Barriers and facilitators to program engagement

Camp feature	Supports engagement	Barrier to engagement
Family-level factors		
Child-level factors	Child interest/enjoyment of program, peer attendance	Resistance to attending, child medical/health conditions
Parent-level factors	Trust and familiarity with staff, family ability to transport children	Program appeal/convenience, poor communication with parents, content/curriculum not clear/reflecting parent values
Program-level factors		
Accessibility & convenience	Convenient schedule/location, flexible attendance, familiar environment, camp hours suit work, simple sign-up, transportation provided, affordable price, spaces available.	High cost, camp size, inconvenient hours, limited/boredom in activities, managing attendance around work, lack of spaces, finding an ideal program, managing multiple camps.
Program design & quality	Appropriate age groups, engaging/special-interest activities, healthy food provided, programs meeting family needs.	Poor or no food/unhealthy food, inadequate learning or PA opportunities.
Staffing & environment	Adequate staff numbers, trained/experienced staff, staff who interact positively with children, effective behavior management.	Negative staff interactions, ineffective behavior management, child injuries.

Note. PA: Physical activity.

## Abbreviations

MVPA: Moderate-to-vigorous physical activity
PA: Physical activity
SDC: Summer day camp
